# Immunology of Cell Death in Cancer Immunotherapy

**DOI:** 10.3390/cells10051208

**Published:** 2021-05-15

**Authors:** Lorenzo Galluzzi, Abhishek D. Garg

**Affiliations:** 1 Department of Radiation Oncology, Weill Cornell Medical College, New York, NY 10065, USA; 2 Sandra and Edward Meyer Cancer Center, New York, NY 10065, USA; 3 Caryl and Israel Englander Institute for Precision Medicine, New York, NY 10065, USA; 4 Department of Dermatology, Yale School of Medicine, New Haven, CT 06520, USA; 5 Université de Paris, 75006 Paris, France; 6 Cell Stress & Immunity (CSI) Lab, Department for Cellular & Molecular Medicine (CMM), KU Leuven, 3000 Leuven, Belgium

**Keywords:** immuno-oncology, immune-checkpoint blockers, necroptosis, apoptosis, inflammation, biomarkers

Over the last two decades, a large volume of studies has established that dying and dead cancer cells exert a potent immunomodulatory effect on their immediate microenvironment, which has a major influence on the anticancer immunity [1,2,3,4,5,6]. Cancer cell death can be driven by the harsh conditions that exist in the tumor microenvironment (TME) [7], as well as by conventional cytotoxic therapies (e.g., chemotherapy, targeted therapy, and radiotherapy) [8,9], and various immunotherapies that operate indirectly by (re-)activating the host immune system against malignant cells (e.g., immune checkpoint inhibitors (ICIs), adoptive T cell therapy (ACT), oncolytic viruses, and chimeric antigen receptor (CAR) T cell therapy) [10,11,12,13]. Cancer cell death can occur via accidental (e.g., necrosis) or (more frequently) regulated (e.g., apoptosis, necroptosis, pyroptosis, or ferroptosis) forms of death that, depending on a large panel of variables, can either promote or inhibit anticancer immunity [14,15,16]. Along similar lines, the precise pathway of cell death at play in malignant cells responding to therapy depends on a myriad of factors, including (but not limited to): (1) treatment dosage and therapeutic schedule [17], (2) cell-intrinsic susceptibility or resistance mechanisms (be they genetic or epigenetic), including the proficiency of stress-responsive pathways such as the endoplasmic reticulum (ER) stress response, the integrated stress response (ISR), and autophagy [18], (3) microenvironmental conditions (e.g., hypoxia and acidosis) [19,20], as well as (4) the ability of treatment to reach malignant cells across various physiochemical barriers (e.g., vasculature, fibrotic tissue, and stroma) within a dysregulated TME [21,22,23,24]. As an additional layer of complexity, multiple cell death pathways are likely to co-exist in tumors responding to treatment [25,26].

Irrespective of the aforementioned complexities, cancer cell death has a major influence on the TME, because dying/dead cancer cells actively secrete or passively release a plethora of bioactive factors, including (but not limited to): (1) reactive oxygen species [27]; (2) cytokines such as type I interferon (IFN), interleukin 1 beta (IL1B, best known as IL-1β), interleukin 6 (IL6), IL33, tumor necrosis factor (TNF), and transforming growth factor beta 1 (TGFB1, best known as TGF-β) [28]; (3) chemokines such as C-C motif chemokine ligand 2 (CCL2), C-X-C motif chemokine ligand 1 (CXCL1), CXCL2, CXCL3, CXCL8, CXCL9, CXCL10, CXCL11, and C-X3-C motif chemokine ligand 1 (CX3CL1) [29]; (4) small metabolites including ATP and other nucleic acids, lipids, and amino acids [30]; (5) mitochondrial components including mitochondrial DNA [31,32]; and (6) various other cellular proteins that are normally less visible to the immune system, such as surface-exposed calreticulin (CALR) or extracellular high mobility group box 1 (HMGB1) [33]. Together with the antigenic profile of dying cells and the immunological baseline configuration of the TME [34,35,36,37,38], these factors regulate the immunological impact of cancer cell death on the TME [39,40,41,42,43]. Notably, a spatiotemporally defined combination of cytokines, chemokines, metabolites, and other damage-associated molecular patterns (DAMPs) emitted by dying cancer cells can support immune reactions that drive cancer-specific T cell immunity, thereby enforcing durable tumor regression and promoting the establishment of immunological memory against malignant cells of the same type [44,45,46,47,48]. Such immunologically active variants of cell death have been named either “immunogenic cell death” (ICD) or “inflammatory cell death”, and their regulation involves a variety of variables beyond DAMP emission, including the antigenic determinants of dying cells and the ability of the TME to enable the priming and execution of adaptive immune responses [14,29,49,50].

Recently, considerable effort has been devoted to understanding the molecular and cellular mechanisms through which the innate and adaptive immune system perceive and decode the complex signals emitted by dying cancer cells [51]. In “Immunology of Cell Death in Cancer Immunotherapy” (a Special Issue of *Cells*), a panel of top-standing scientists working on the immunology of cell death gathered to foster a comprehensive discussion on how the immunological features of dying and dead cancer cells can be exploited for improving cancer immunotherapy. Collectively, the ten contributions in “Immunology of Cell Death in Cancer Immunotherapy” provide a comprehensive description of the main molecular, immunological, and therapeutic aspects of cancer cell death immunology and immunotherapy.

Shortly after cancer cell death, the recruitment of innate immune cells and their ability to take up particulate material (i.e., phagocytosis) enables the first contact between cell corpses and the immune system [52]. The duration and nature of these contacts has a major impact on the activation vs. inhibition of anticancer immunity. In their review article [53], Gadiyar et al. (Raymond B. Birge’s lab) discuss the chemo-attraction of phagocytic cells by dying cancer cells, as well as the immunological consequences of efferocytosis and how cancer cells harness these proximal events in support of tumor progression and immune escape, culminating with a description of therapeutic strategies to overcome these pro-tumorigenic processes. Translating such fundamental (in vitro) insights into tangible outcomes is a challenging task. Indeed, biological context often constitutes a strong bottleneck in the conceptualization and preclinical modeling of therapeutic regimens. In their research paper [54], Flieswasser and colleagues (Julie Jacobs’s lab) evaluated the differential in vitro vs. in vivo capacity of various chemotherapeutics (docetaxel, carboplatin, cisplatin, oxaliplatin, and mafosfamide) to induce ICD in pulmonary malignancies. Interestingly, the authors demonstrate that while most of these chemotherapies induce multiple hallmarks of ICD (e.g., DAMP emission) in vitro, they did not always stimulate anticancer immunity in vivo, in gold-standard mouse vaccination models [55]. This work underscores the continuous challenge of translating in vitro observations based on surrogate ICD biomarkers into an anticancer immune output in vivo. Another key obstacle for ICD-inducing chemotherapeutics is cancer cell-intrinsic resistance to treatment. In their original article [56], Kopecka and colleagues (Chiara Riganti’s lab) explored chemotherapy resistance driven by active drug export by P-glycoproteins (Pgp). They found that pharmacological Pgp inhibitors such as tariquidar or an R-3 compound (*N*,*N*-bis(alkanol)amine aryl ester derivative) can be used in combination with doxorubicin to rescue ICD in Pgp-expressing, doxorubicin-resistant cancer cells. ICD resistance can also originate from cancer cell-extrinsic factors linked to the immunological configuration of the TME. Common mechanisms of TME-associated resistance to ICD include hypoxia, acidosis, vasculature disorganization, and stromal/myeloid cell immunosuppressive signaling [57]. In their review article [58], Vito et al. (Karen Mossman’s lab) summarize current knowledge on hypoxia-driven immune escape, as they discuss the Janus face of hypoxia in the regulation of tumor-targeting immunity.

Over the past few years, substantial evidence has accumulated in support of combining conventional therapies (including, but not limited to, ICD inducers) with ICIs to achieve potent anticancer immune responses in specific oncological settings [1,59,60,61,62]. In their review article [63], Dosset and co-authors (Lionel Apetoh’s lab) discuss the prospects of combining immunogenic anticancer therapies with ICIs toward superior clinical efficacy. Specifically, they discuss how conventional anticancer agents may create an immune infiltrate that is permissive of ICI activity, depending on the sequence, dose, and duration of treatment. In this context, Rossi and co-investigators (Laura Bracci’s lab) [64] compared the ability of cyclophosphamide or cisplatin to synergize with ICIs targeting programmed cell death ligand 1 (PDCD1LG1, best known as PD-L1), or programmed cell death 1 ligand 2 (PDCD1LG2, best known as PD-L2). They discovered that the success of these combinations (i.e., magnitude and duration of response) is contingent upon the immunogenic potential of chemotherapy and the nature of the tumor. Altogether, these results highlight the importance of cancer cell-intrinsic immunogenicity and the choice of chemotherapy in maximizing the success of ICIs [65]. The field of cancer immunotherapy is also moving toward “smart” therapeutic modalities, wherein multiple immunostimulatory modalities can be integrated within a single therapeutic platform, such as oncolytic virotherapy or nanotherapy. In their original contribution [66], Heinio et al. (Akseli Hemminki’s lab) explored a novel way of breaking the resistance of the TME to tumor-targeting immunity by harnessing a cytokine-armed oncolytic adenovirus: Ad5/3-d24-E2F-hTNFa-IRES-hIL2 (also known as TILT-123 and OAd.TNFa-IL2). They found that the cytokine-armed oncolytic adenovirus exploits DAMP- and microbe-associated molecular patterns (MAMP)-driven immune responses in support of tumor regression. These findings illustrate the significant potential of rationally engineered oncolytic viruses for cancer immunotherapy [67].

Despite the tremendous progress achieved over the past decade, the clinical translation of the preclinical concepts linked to ICD in cancer therapy, discussed here, remains a major challenge [68]. In this context, biomarker-driven approaches stand out as a promising strategy to identify cancer patients at increased likelihood to respond to specific ICD inducer-based therapies. In their review article [69], Sprooten and co-workers (Abhishek D. Garg’s lab) summarize the current knowledge on the role of necroptosis (a regulated variant of necrosis) in immuno-oncology and cancer immunotherapy. Specifically, they discuss recent preclinical studies on necroptosis-driven anticancer immunity and compensatory immunosuppression, as well as contradicting clinical evidence based on ambiguous necroptosis biomarkers. Vaes and colleagues (Dirk De Ruysscher’s lab) [70] provide a comprehensive summary of biomarkers associated with radiotherapy-induced ICD, largely focusing on clinical literature as a means to capture the current interest on prognostic or predictive biomarkers associated with radiotherapy-induced ICD-mediated immunity. While these studies largely discuss genomic, transcriptomic, or proteomic biomarkers, other factors may have prognostic or predictive value in immuno-oncology, including metabolic and epigenetic variables [71]. In their research paper [72], Zhou and collaborators (Udo S. Gaipl’s lab) attempted to decipher the prognostic impact of long noncoding RNAs (lncRNAs) in the context of immuno-oncology clinical trial data. Through a series of bioinformatics analyses, Zhou et al. uncovered a 15 lncRNA signature with predictive value in advanced melanoma patients treated with PD-1-targeting ICIs. These results highlight the underappreciated role of lncRNAs as immuno-oncology biomarkers.

In summary, this Special Issue of *Cells* underscores the extreme complexity associated with translating preclinical findings into clinically actionable approaches for treatment or patient stratification, with a major focus on tumor context. We believe that the issues associated with current mouse models of cancer, specifically their limited ability to recapitulate the heterogeneity of human malignancies and natural immune variations [73], as well as the lack of reliable prognostic and predictive biomarkers, constitute a significant bottleneck for the clinical translation of ICD-associated therapy. We surmise that well-designed reverse translational approaches may be instrumental for the development of the next generation of immuno-oncology agents.

## Data Availability

Not available.
